# *Mycoplasma conjunctivae* in domestic small ruminants from high mountain habitats in Northern Spain

**DOI:** 10.1186/1746-6148-9-253

**Published:** 2013-12-13

**Authors:** Xavier Fernández-Aguilar, Óscar Cabezón, Ignasi Marco, Gregorio Mentaberre, Joachim Frey, Santiago Lavín, Jorge R López-Olvera

**Affiliations:** 1 Servei d’Ecopatologia de Fauna Salvatge (SEFaS), Departament de Medicina i Cirurgia Animals, Facultat de Veterinària, Universitat Autònoma de Barcelona, 08193 Bellaterra, Spain; 2 Centre de Recerca en Sanitat Animal (CReSA), UAB-IRTA, Campus de la Universitat Autònoma de Barcelona, 08193 Bellaterra Barcelona, Spain; 3 Institute of Veterinary Bacteriology, University of Bern, Länggass-Strasse 122, CH-3012 Bern, Switzerland

**Keywords:** Goat, Sheep, Infectious keratoconjunctivitis, Mycoplasma conjunctivae, Spain, Pyrenees, Cantabrian mountains

## Abstract

**Background:**

Infectious keratoconjunctivitis (IKC) is a clinical condition affecting eyes of domestic and wild *Caprinae* worldwide, and *Mycoplasma conjunctivae* is considered the primary causative agent of IKC in sheep, goats and wild *Caprinae*. Domestic ruminants from high mountain habitats share grazing areas with wild mountain ungulates, such as chamois (*Rupicapra* spp.), Alpine ibex (*Capra ibex*) and European mouflon (*Ovis aries musimon*), and domestic sheep seem to act as *M. conjunctivae* reservoir. In this study, the presence of *M. conjunctivae* in domestic sheep and goats from the two main mountain ranges of Northern Spain, the Pyrenees and the Cantabrian Mountains, has been investigated.

**Results:**

Eye swabs were obtained from 439 domestic small ruminants selected from flocks that seasonally graze in alpine meadows during three consecutive years (2011-2012-2013). Seventy-nine out of the 378 domestic sheep (20.9%) tested positive to a *M. conjunctivae* specific real time-PCR (rt-PCR) in at least one eye, whereas all the 61 sampled domestic goats were negative. Statistically significant higher prevalence and higher proportion of infected flocks (P < 0.001) was observed in the Pyrenees (25.7%; 12 flocks out of 13), where *M. conjunctivae* is widespread and probably endemic in domestic sheep, than in the Cantabrian Mountains (7.8%; one flock out of six). Twenty-five sheep (three from the Pyrenees and 22 from the Cantabrian Mountains) which showed clinical signs consistent with infectious keratoconjunctivitis (IKC) were negative by rt-PCR. In contrast, 62 out of the 71 (87.3%) *M. conjunctivae*-positive sheep from the Pyrenees and the eight positive sheep from the Cantabrian Mountains were asymptomatic.

**Conclusions:**

This study provides rt-PCR-based evidences of *M. conjunctivae* maintenance in domestic sheep, as well as a relationship between prevalence in domestic sheep and previously reported *M. conjunctivae* and IKC in wild ruminants. Domestic goats do not seem to play an important role in the epidemiology of *M. conjunctivae* in alpine habitats from Northern Spain.

## Background

Infectious keratoconjunctivitis (IKC) is a clinical condition affecting eyes of domestic and wild *Caprinae* worldwide. Several infectious agents such as *Mycoplasma conjunctivae*, *Chlamydophila psittaci* or *Moraxella ovis* (formerly *Branhamella ovis*) have been isolated from eyes of small ruminants affected by IKC [1]. However, *M. conjunctivae* is considered the primary causative agent of IKC in sheep, goats and wild *Caprinae*[1-4]. Susceptibility to *M. conjunctivae* infection differs among host species. While in sheep and goats IKC usually appears in form of transitory blindness causing little concern and economic consequences, pathogenicity to wild species is generally high though variable, causing outbreaks with morbidity and mortality up to 30% [1].

The epidemiology of *M. conjunctivae* is particularly worth investigating in mountain habitats, where domestic ruminants share grazing areas with susceptible wild mountain ungulates during late spring (May-June) to early fall (September-October), such as chamois (*Rupicapra* spp.), Alpine ibex (*Capra ibex*) and European mouflon (*Ovis aries musimon*) [1,5]. Interspecific transmission may occur [6], and domestic sheep seem to play a key role as a reservoir host for *M. conjunctivae* in such a complex scenario of host interaction [7,8]. Several outbreaks of IKC have been described in domestic sheep and goats worldwide [3,9-11], but few active surveillance studies have been conducted in small domestic ruminants, particularly in high mountain habitats.

**Figure 1:**
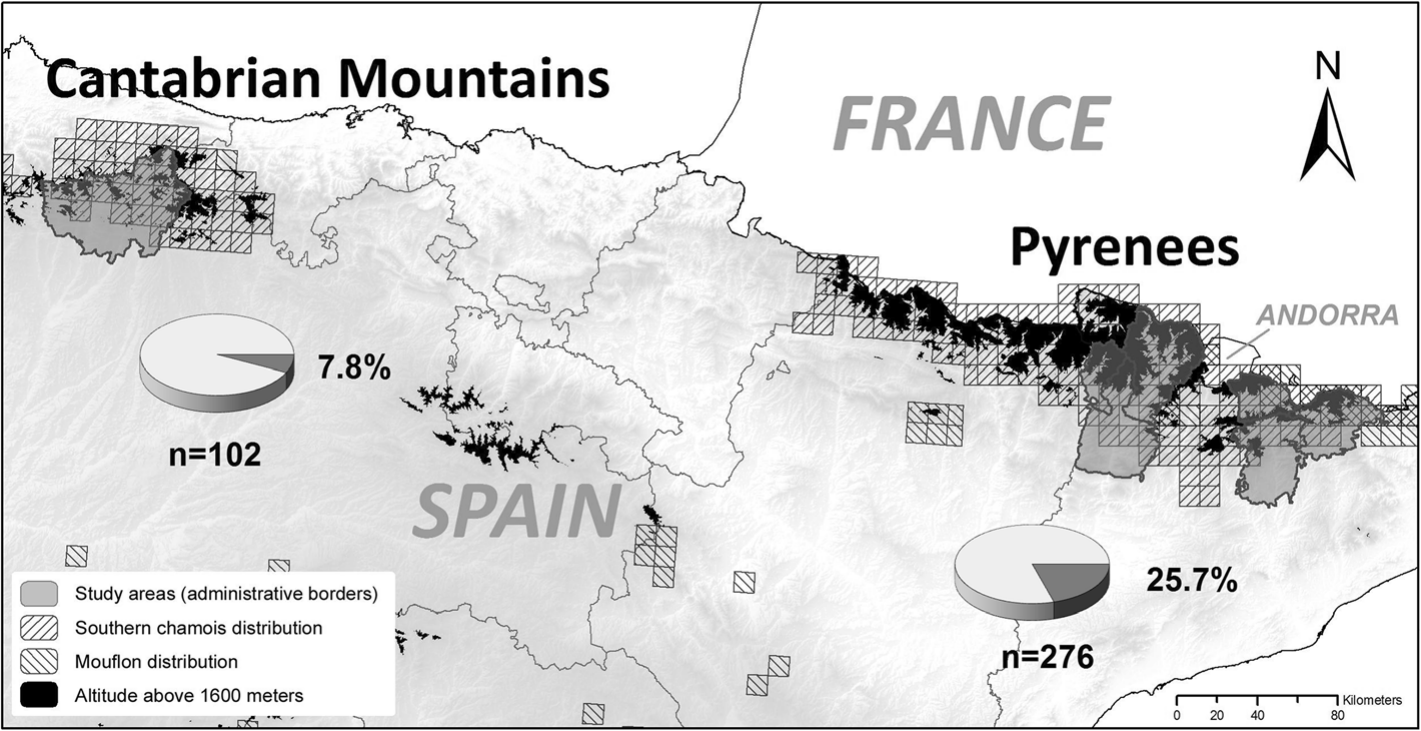
**Pie charts showing the prevalence of *M. conjunctivae *in domestic sheep in the Cantabrian Mountains and the Pyrenees.** Altitude above 1600 meters is shown in black. Borders for the regions where the sampled flocks graze in alpine meadows are shown in darker grey. Southern chamois and mouflon distribution squares correspond to a 10x10 Km grid.

In this study, the presence of *M. conjunctivae* in domestic sheep and goats from the two main mountain ranges of Northern Spain, the Pyrenees and the Cantabrian Mountains, has been investigated (Figure 1). *M. conjunctivae* and IKC outbreaks have been reported in Pyrenean chamois (*Rupicapra pyrenaica pyrenaica*) in the Pyrenees [5,12,13], but not in Cantabrian chamois (*Rupicapra pyrenaica parva*) from the Cantabrian mountains. Therefore, both study areas represent two different epidemiological scenarios.

## Methods

Conjunctival swabs were obtained from below the nictitating membrane from both eyes in 439 physically restrained small ruminants (19 flocks; 378 sheep, 61 goats) from the Catalan Pyrenees (Eastern and Central Pyrenees; 13 flocks; 276 sheep and 24 goats) and the southern side of the Cantabrian Mountains (6 flocks; 102 sheep and 37 goats). Flocks that graze in alpine meadows were selected according its potential contact with wild hosts susceptible to *M. conjunctivae* infection, namely Pyrenean chamois and European mouflon, in the National Game Reserves (NGR) of Freser-Setcases, Cadí, Cerdanya, and Alt Pallars in the Pyrenees, and Cantabrian chamois in the National Park of Picos de Europa and NGR of Mampodre and Riaño in the Cantabrian Mountains. Handling procedures were designed to reduce stress and health risks for subjects, according to European (86/609) and Spanish laws (R.D. 223/1988, R.D.1021/2005), and current guidelines for ethical use of animals in research [14]. A mean number of 20 sheep and goats were randomly sampled within each flock from November to May in three consecutive housing periods (2010–2011, 2011–2012 and 2012–2013) (Table 1). Two flocks in each region were sampled in at least two periods. All the goats were sampled in mixed goat and sheep flocks, except for 20 goats from a goat flock in the Cantabrian Mountains. Clinical signs compatible with IKC were recorded at sampling and swabs were stored at -20ºC until analyzed.

**Table 1:** **Results of *Mycoplasma conjunctivae *** **prevalence assessed by rt-PCR (as described in [**[15]**])**

**Sampling period**	**2010-2011**		**2011-2012**		**2012-2013**		**Total**		
	**Prevalence**	**Flocks**	**Prevalence**	**Flocks**	**Prevalence**	**Flocks**	**Prevalence**	**CI 95%**	Flocks*
**Goats**									
Pyrenees	NS	NS	0.0 (0/10)	0/5	0.0 (0/14)	0/3	0.0 (0/24)	-	0/7
Cantabrian Mountains	0.0 (0/5)	0/1	0.0 (0/4)	0/1	0.0 (0/28)	0/2	0.0 (0/37)	-	0/2
Total	0.0 (0/5)	0/1	0.0 (0/14)	0/6	0.0 (0/42)	0/5	0.0 (0/61)	-	0/9
**Sheep**									
Pyrenees	NS	NS	26.1 (29/111)	6/7	25.4 (42/165)	7/8	25.7 (71/276)	20.5 – 30.9	12/13
Cantabrian Mountains	25.0 (3/12)	1/2^†^	0.0 (0/18)	0/2	6.9 (5/72)	1/4^†^	7.8 (8/102)	2.6 – 13.0	1/5
Total	25.0 (3/12)	1/2	22.5 (29/129)	6/9	19.8 (47/237)	8/12	20.9 (79/378)	16.8 – 25.0	13/18
**Total**	17.6 (3/17)	1/2	20.3 (29/143)	6/10	16.8 (47/279)	8/13	18.0 (79/439)	14.4 – 21.6	13/19

NS: Not sampled.

*Total number of flocks may no coincide with the sum of all the periods because some of the flocks were re-sampled in more than one period.

^†^In the Cantabrian Mountains there was a single *M. conjunctivae*-positive flock, sampled in both periods (2010–2011 and 2012–2013).

During the study period, no outbreak of IKC was detected neither in domestic ruminants or wild mountain ungulates in the study area, though IKC cases in Pyrenean chamois are observed every year (Fernández-Aguilar *et al*., in prep.).

At the laboratory, swabs were thawed and mixed with 0.5 ml of lysis buffer (100 mM Tris–HCl, pH 8.5, 0.05% Tween 20, 0.24 mg/ml proteinase K) in microcentrifuge tubes and cells were lysed for 60 minutes at 60°C and 15 minutes at 97°C. The presence of *M. conjunctivae* was determined by a TaqMan real-time PCR (rt-PCR) with an exogenous external positive control in each reaction, as previously described [15]. For analysis of *Mycoplasma agalactiae* as a potential cause of IKC, the real time PCR method based on the cytadhesin P40 [16] was used as described [17].

Chi-square tests were performed in order to detect statistically significant differences in *M. conjunctivae* prevalence both at flock and individual level according to area and clinical status, using the PROC FREQ of the SAS® 9.1.3 System for Windows (SAS Institute, Cary, NC, USA).

## Results

*Mycoplasma conjunctivae* was detected in at least one eye of 79 out of the 378 domestic sheep (20.9%) analyzed. Both individual and flock prevalence were significantly higher (p < 0.001) in the Pyrenees (25.7%; 71 of 276 sheep; 12 positive flocks out of 13) than in the Cantabrian Mountains (7.8%; 8 of 102 sheep; one positive flock out of six) (Table 1 and Figure 1). Mean prevalence within infected sheep flocks was 29.9% (range 6.7% to 65%) in the Pyrenees and 32% in the only positive flock from the Cantabrian Mountains. The flocks sampled in two periods did not change their status regarding the presence of *M. conjunctivae* (one positive and one negative flock in each region). Moreover, the five sheep sampled twice in the *M. conjunctivae*-positive flock from the Pyrenees kept their status (two positive and three negative sheep) in both sampling periods which were separated by 12 months.

**Figure 2:**
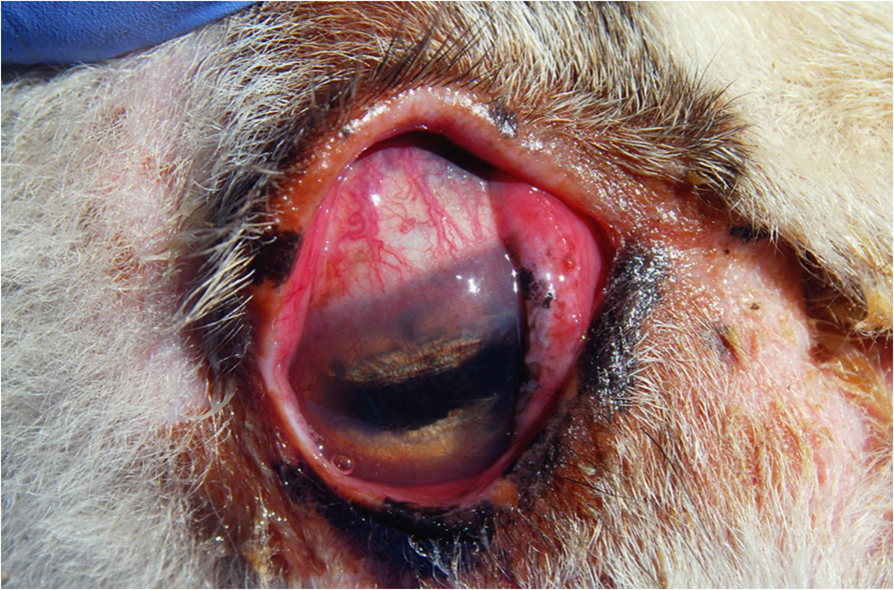
One of the more severe IKC cases observed during the study period, eye of a domestic sheep from the Pyrenees showing epiphora, conjunctival hyperemia, peripheral corneal edema, and neovascularization.

Clinical signs consistent with IKC, such as ocular discharge, epyphora, mild conjunctivitis and/or corneal opacity were registered in 4.3% (12/276) of the examined domestic sheep from the Pyrenees and in 21.6% (22/102) in the Cantabrian Mountains (Figure 2). Most of the cases of IKC in sheep from the Pyrenees were associated with the presence of *M. conjunctivae* (75%), whereas none of the 22 sheep and three goats that showed clinical signs in Cantabrian Mountains had *M. conjunctivae* in their eyes. As *Mycoplasma agalactiae* has been reported to be a mycoplasmal cause of IKC in domestic ruminants [18], samples of these sheep were also analyzed by real time PCR for the presence of this pathogen. This analysis did not detect *M. agalactiae* in the eye swabs of these animals. In contrast, 62 out of the 71 (87.3%) *M. conjunctivae*-positive sheep from the Pyrenees and the eight positive sheep from the Cantabrian Mountains were asymptomatic. In most flocks, IKC was unnoticed by the owners, as clinical cases were found to be occasional and mild. The clinical signs of the two sheep from the Pyrenees which were positive to *M. conjunctivae* in two samplings periods evolved from lachrymation and bilateral conjunctivitis in the first sampling to apparently healthy eyes in the second sampling in one sheep, and conversely for the other sheep.

*M. conjunctivae* was not detected in any of the goats sampled in both study areas (Table 1), although three goats from one flock in the Cantabrian Mountains showed mild clinical ocular signs.

## Discussion

The high prevalence of *M. conjunctivae* in domestic sheep from the Pyrenees and its maintenance over sampling periods (Table 1) indicate that *M. conjunctivae* infection is widespread among domestic sheep in the Pyrenees, endemic and self-maintained within domestic sheep flocks. This agrees with previous data on *M. conjunctivae* prevalence (25.8%) in domestic small ruminants from Central Pyrenees [12] and the wide distribution of *M. conjunctivae* infection in domestic sheep throughout Europe [4,7]. In contrast, the lower prevalence found in the Cantabrian Mountains and the fact that *M. conjunctivae* was detected in only one flock in this region suggest that *M. conjunctivae* is currently less common in this area. Moreover, the only *M. conjunctivae*-positive flock in the Cantabrian Mountains seasonally migrates to Caceres (South-Western Spain) in winter. Hence, the origin of *M. conjunctivae* in this flock is unclear and could lead to an overestimation of the endemic situation of *M. conjunctivae* in this region. Furthermore, the occurrence of *M. conjunctivae* relative to IKC cases in the two study areas strongly differed. Whereas there is a good agreement between IKC cases and the presence of *M. conjunctivae* in the Pyrenees, this is not the case in the Cantabrian Mountains.

The prevalence of *M. conjunctivae* among asymptomatic sheep was higher than previous data obtained by traditional culture methods [19]. However, strain pathogenicity or individual host factors such as immunity may influence the outcome of clinical disease. Spontaneous clinical recoveries and relapses are a common feature of IKC in sheep [4], which agrees with the clinical evolution of the two positive sheep from the Pyrenees sampled twice, further suggesting the role of sheep as a maintenance host of *M. conjunctivae* in this area, as previously suggested in Switzerland assessed by serology [7] and Central Pyrenees [12].

Several IKC outbreaks caused by *M. conjunctivae* have been reported in domestic goats [3,9,10], and IKC has been experimentally reproduced in goats by inoculating *M. conjunctivae* previously isolated from a goat with IKC [2]. However, the absence of *M. conjunctivae* occurrence in goats in this study, even in mixed flocks with high prevalence of *M. conjunctivae* in sheep, suggests a lower susceptibility to *M. conjunctivae* infection or a host specificity of the strains circulating in the area among domestic sheep. Therefore, domestic goats do not seem to contribute to *M. conjunctive* epidemiology in nonepidemic IKC in mountain habitats from Northern Spain. The finding of sheep and goats negative for *M. conjunctivae* and *M. agalactiae* but showing clinical signs consistent with IKC suggests a possible implication of other pathogens, such as *Chlamydophila psittaci*, *Moraxella ovis*, or *Listeria monocytogenes* previously detected in domestic small ruminants with IKC [1,19]. The higher occurrence of *M. conjunctivae*-negative sheep and goats with clinical signs of keratoconjunctivitis in the Cantabrian Mountains suggests that other pathogens may be more relevant than *M. conjunctivae* for keratoconjunctivitis in this region. Although *M. conjunctivae* is considered the main etiological agent of IKC outbreaks in both domestic small ruminants and wild mountain ruminants [1,2,11], pathogens or conditions associated with nonepidemic IKC warrant further research, as previously suggested in wild mountain ungulates [20].

The widespread and consistent presence of *M. conjunctivae* in domestic sheep from the Pyrenees and the less relevant role of this pathogen in the Cantabrian Mountains seem to correspond to previous reports of IKC in wild sympatric susceptible hosts, such as European mouflon and Pyrenean chamois in the Pyrenees [1,5,12,13], but not in Cantabrian chamois from the Cantabrian Mountains. Phylogenetical analyses of *M. conjunctivae* strains circulating in domestic sheep and wild mountain ruminants would help clarifying the specific role of different host species in the epidemiology of IKC from the studied areas, particularly in the Pyrenees.

## Conclusions

This study provides rt-PCR-based evidence of *M. conjunctivae* maintenance in domestic sheep, as well as a relationship between prevalence in domestic sheep and previously reported *M. conjunctivae* and IKC in wild ruminants. This finding adds new relevant information into the epidemiology of *M. conjunctivae* in the domestic-wildlife interface. Domestic goats do not seem to play an important role in the epidemiology of *M. conjunctivae* in alpine habitats from Northern Spain.

## Competing interests

The authors declare no competing interests.

## Authors’ contributions

XF and OC collected samples and performed the rt-PCR assays. XF drafted the manuscript. OC conceived the study and critically revised the manuscript. IM, GM and SL provided access to and collected samples, gave technical and material support, and critically revised the manuscript. JF provided the controls for and coordinated the rt-PCR assays, participated in the study design and critically revised the manuscript. JRLO acquired funding, elaborated the study design, collected samples, coordinated the analyses and participated in the elaboration of the draft. All authors read and approved the final manuscript.
